# Don’t overthink it: The paradoxical nature of expertise for the detection of errors in conceptual business process models

**DOI:** 10.3389/fnins.2022.982764

**Published:** 2022-11-24

**Authors:** Karl-David Boutin, Christopher Davis, Alan Hevner, Pierre-Majorique Léger, Elise Labonte-LeMoyne

**Affiliations:** ^1^Department of Information Technologies, HEC Montréal, Montreal, QC, Canada; ^2^School of Information Systems and Management, University of South Florida, Tampa, FL, United States

**Keywords:** conceptual modeling, process modeling, eye tracking, attentional characteristics, expertise, business process management, BPMN, business analysts

## Abstract

Business process models are widely used artifacts in design activities to facilitate communication about business domains and processes. Despite being an extensively researched topic, some aspects of conceptual business modeling are yet to be fully explored and understood by academicians and practitioners alike. We study the attentional characteristics specific to experts and novices in a semantic and syntactic error detection task across 75 Business Process Model and Notation (BPMN) models. We find several intriguing results. Experts correctly identify more error-free models than novices, but also tend to find more false positive defects. Syntactic errors are diagnosed faster than semantic errors by both groups. Both groups spend more time on error-free models. Our findings regarding the ambiguous differences between experts and novices highlight the paradoxical nature of expertise and the need to further study how best to train business analysts to design and evaluate conceptual models.

## Introduction

Business process modeling activities are used to communicate and share knowledge, design and improve processes, and re-design decisions in organizations (Becker et al., 2004; Indulska et al., 2009; Recker et al., 2012). While always considered a key tool in information systems (IS) development, business process models are becoming more widely studied in the last decade. Evidence shows that despite the effort of the academic community, practitioners (e.g., business analysts) still struggle with some aspects of conceptual modeling, such as the standardization of modeling notations and methodologies (Eikebrokk et al., 2008) and the emerging requirements of an increasingly digital world (Recker et al., 2021). Furthermore, the focus of the academic community does not always correspond with the practitioners’ needs (Indulska et al., 2009). Areas of interest such as individuals’ performance and practitioners’ training seem to be of little interest to the research community compared to other elements, such as modeling grammar and method (Wand and Weber, 2002; Indulska et al., 2009; Jabbari Sabegh et al., 2017). This creates a significant gap in the literature as the lack of knowledge on the nature of expertise in conceptual modeling is a hindrance to the improvement of business analysts’ training curriculum (Davis et al., 2018). The use of a widely accepted visual notation, such as Business Process Model and Notation (BPMN) or Universal Modeling Language (UML), which has its own set of rules and constraints, allows the model to be understood by people across different departments and organizations (Davis et al., 2018). Yet, when using a visual notation, it can still be hard to fully grasp the model and it is almost impossible to design a flawless model without any confusing ambiguities (Figl, 2017). To refine the notations and to improve the training of future business analysts, we need to understand how, on a cognitive level, business analysts read and comprehend graphical process models (Hungerford et al., 2004).

To better understand expert modelers’ heuristics, the main objective of the study is to probe the difference in cognitive processing between experienced business analysts and novices while reading and diagnosing errors in conceptual models. By exploring and understanding the differences between neophytes and more experienced modelers, we hope to highlight what can be considered ‘best practices’ in deciphering models and at the same time identify some key limitations of visual notations. To do so, we use the concept of expertise to compare two groups of modelers in order to identify the skill-based adaptations that differentiate novice and expert designers (Davis et al., 2018). Understanding the heuristics of the experts in an error detection task will allow us to adapt the training curricula to facilitate the development of future business analysts.

Several researchers have explored the variations between novice and expert dichotomy in conceptual modeling (Shanks, 1997; Yusuf et al., 2007; Recker and Dreiling, 2011; Koschmider et al., 2015). However, we are aware of no studies exploring the repertoire of skills, or “competencies,” outlined in Bassellier et al. (2003), to assess their interdependence and capacity to differentiate novices from more experienced business analysts. The skills and abilities that differentiate experienced business analysts from novices, covering a broad spectrum, make the identification of success factors in conceptual modeling difficult.

This research strives to deepen understanding of the sometimes simplistic expert-novice dichotomy evident in prior studies, using eye-tracking instruments to capture an objective measurement of the actual behavior of our business analysts via their visual attention (Bera et al., 2019). Our efforts to more fully articulate the expert-novice dichotomy also strives to identify and ameliorate limitations in the literature. Specifically, in this work, we considered semantic and syntactic error detection tasks by scrutinizing the differences between successful anomaly detection and unsuccessful diagnostics.

Overall, we were surprised to find that our results suggest few statistically significant differences between novices and experts. In fact, experts tend to detect more non-existent anomalies (false positives) in error-free models than novices. However, experts correctly diagnose error-free stimuli more efficiently. Syntactic errors tended to be diagnosed more quickly than semantic errors and models without any errors generally took more time to diagnose than other models.

The paper is organized as follows: first, a literature review of the main concepts, starting by exploring the concept of conceptual modeling, then examining prior studies regarding expertise, and finally delving into the use of eye-tracking devices to capture visual attention. The methodology, instruments, and measures used in the experiment are explained. The results are then listed and analyzed, before concluding the paper with insights for future research and applied practice.

## Literature review

### Conceptual modeling

Conceptual modeling is a complex activity (Wand and Weber, 2002; Nelson et al., 2012), essential to the design of IT artifacts (Davis et al., 2018). More than just a tool to facilitate comprehension of business processes (Figl, 2017), conceptual models are used, among other things, as a communication medium between users and developers, and to help business analysts understand business domains (Kung and Solvberg, 1986; Parsons and Cole, 2005; Moody, 2009; Bavota et al., 2011). They also play an important part in business process transformation, since they greatly facilitate the investigation of problems and limitations in organizations (Liberatore et al., 2000). Conceptual models are also used as a bridge between the business and IT actors, allowing them to understand each other easily and, thus, allowing them to work together on improving the business processes of the organization (Birkmeier et al., 2010).

By tapping into two-dimensional graphic space, diagrams and models allow the organization of information by location, rather than having to follow a linear path like a textual representation (Larkin and Simon, 1987). This means that the relevant information is usually located in one place, which makes implicit information more obvious and models more concise than textual cases (Moody, 2009). This type of representation allows the business analysts to understand a situation, or problem, by crossing the diagram quickly, focusing on the different groups of information, rather than deciphering a text in their search for relevant elements (Larkin and Simon, 1987). Furthermore, the use of pictures has been shown to facilitate the acquisition and retention of information more readily than through the use of printed items.

Visual notations are composed of *visual syntax*, encompassing the visual vocabulary, which is the set of symbols and the visual grammar, and *visual semantics*, which give meaning to the different symbols and to their relationship (Moody, 2009; Nelson et al., 2012; Davis et al., 2018). However, while most studies concentrate on the effect of semantics on the comprehension of a model, for example by studying the level of abstraction of labels (Mendling and Strembeck, 2008; Mendling et al., 2010; Figl and Strembeck, 2015), few researchers have examined the effect of syntactic rules or offered syntactic guidelines (Moody, 2009; Figl, 2017). This represents a significant gap in the literature, since syntactic differences between notations are as important, if not more prominent, than semantic variances (Moody, 2009).

The increasing popularity of process modeling in IS has spawned a significant number of notations and techniques to create conceptual models. This has had the effect of increasing the number and disparity of academic and professional formations, each having to choose which notations to teach and how to teach it. Organizations also must choose which notation to use for their process modeling and software suppliers need the follow the demand and supply tools for the most popular notations (Recker and Dreiling, 2007). All those questions create a fertile environment for research. Furthermore, the lack of study comparing the differences between the semantic and syntactic components of visual notations, or simply the effect of the syntactic rules, present another opportunity to contribute to the literature.

While some experiments have compared different notations or presentation mediums in order to identify the one having the most significant effect on comprehension (Recker and Dreiling, 2007; Yusuf et al., 2007; Ottensooser et al., 2012; Rodrigues et al., 2015), others have studied the effect of prior domain or modeling knowledge between users (Bera, 2012; Recker, 2013; Recker et al., 2014; Figl and Laue, 2015; Kummer et al., 2016). However, while there are recommendations on how to create better models, or how to adapt the models to the user’s experience, few recommendations are made on how to improve training curriculums.

In this study, we use the Business Process Model and Notation (BPMN) specification from the Object Management Group. BPMN is widely used in industry and has been the subject of several recent studies on quality issues in organizational process models. An analysis of 585 BPMN process models from six companies identified significant quality concerns on issues of model structure, layout, and labeling (Leopold et al., 2016). Five modeling recommendations are offered that claim to address over 90% of the identified quality issues. An in-depth study of human inspection strategies on BPMN models found a number of important challenges that reduced the effectiveness of finding defects in process models (Haisjackl et al., 2018). Twelve experienced analysts inspected BPMN models of moderate complexity to find syntactic, semantic, and pragmatic defects. Using ‘think-aloud’ research methods, challenges were identified in the areas of lack of domain knowledge, lack of BPMN knowledge, unclear inspection criteria, and many false positives found. However, we found no in-depth studies that use eye tracking technologies to analyze human cognition in the inspection of BPMN models (Batista Duarte et al., 2021).

### The evolving nature of expertise in conceptual modeling

While the criteria to be considered an expert varies widely between fields and professions, since there is no consensus on the definition of expertise (Wineburg, 1998; Davis and Hufnagel, 2007), researchers tend to agree that, usually, experts are faster, more precise and more efficient than novices in their respective field (Speelman et al., 1998; Sonnentag, 2000). The main difference between novices and experts seems to be their organization of knowledge (Herbig and Büssing, 2004). Experts have more detailed and tightly connected schemata (Glaser, 1984; Lurigio and Carroll, 1985), which is defined by Glaser (1984) as the representation of the “knowledge that we experience—interrelationships between objects, situations, events, and sequences of events that normally occur” (Glaser, 1984, p. 100; Wineburg, 1998). Experts, thus, will be able to infer other knowledge from the literal cues in a situation or a problem statement, whereas novices have less sophisticated strategies for using their knowledge to ‘pick up’ such subtle cues (Glaser, 1984; Lurigio and Carroll, 1985). Furthermore, the acquisition of a skill can bring changes to the brain, both by modifying the area of activation when processing a stimulus, to morphological changes increasing the gray matter dedicated to processing the type of stimuli trained for Hill and Schneider (2006). Chess players and radiologists, among other professionals requiring improved perceptual-motor skills, will have a higher performance using lower processing levels than novices, allowing them to perform more difficult discrimination tasks. Per contra, novices tend to use high-level processing, based more on generalizations (Hill and Schneider, 2006).

Evaluating expertise in the conceptual modeling environment is more complex than it seems since working with models require two different kinds of expertise: domain expertise, or expertise related to the semantic component of the models, and modeling expertise, or expertise related to the details of the modeling notations. An expert modeler, well versed in the creation of models using visual notation, may find it quite hard to understand a model depicting a process from a domain on which he doesn’t have any prior knowledge. The opposite is also true; an expert in a domain may have some difficulties reading a conceptual model if he doesn’t know the meaning of the symbols or if he is not used to working with models, even if the process depicted is well known to him.

In prior studies, a multitude of variables has been used to define modeling expertise, or expertise regarding the syntactic component of models, between groups. For example, self-reported measures on modeling familiarity (Reijers and Mendling, 2011; Weitlaner et al., 2013), frequency of work with models (Mendling and Strembeck, 2008; Zimoch et al., 2018b) or objective measures of modeling knowledge (Mendling and Strembeck, 2008; Figl et al., 2013; Recker, 2013; Figl and Laue, 2015) have been used to compare groups of more experienced modelers, or ‘experts’, with novices. Across all of those studies and measures used, the frequent use of flowcharts, prior experience with conceptual modeling (e.g., number of models created or read) and prior training had significant effect on model comprehension, where self-reported measures of knowledge and prior familiarity with modeling didn’t differentiate the participants’ comprehension (Figl, 2017). In conceptual modeling, domain or “semantic” expertise is usually assessed using self-reported measures on perceived domain knowledge (Figl, 2017). Across the experiments that studied the effect of prior domain knowledge on comprehension or performance, no significant effect has been observed.

Rather than studying the difference between expert modelers and novices, where expert modelers have been described in prior experiments with having at least four years of experience as modelers and had contributed to the development of at least ten conceptual models (Shanks, 1997), we focus our attention on the business analysts. Indeed, nowadays the majority of business analysts have to work continuously with conceptual models, whether by creating or reading them, and thus, form the core of the practitioners. Furthermore, in accordance with the concept of IT competence, as defined by Bassellier et al. (2003), business analysts have more IT knowledge – which is the relevant knowledge and the capability to access more IT-related knowledge – and IT experience than novices (Bassellier et al., 2003). Therefore, business analysts, by having more experience with IT projects and by possessing deeper understanding and IT-related knowledge, are better suited as practitioners to use and interact with conceptual models than novices (i.e., individuals who have limited or no experience in situations characteristic of their domain) without any significant IT-related experience or knowledge.

Moreover, Patel and Groen’s distinction of “specific” and “generic” expertise, where “generic” experts have generic knowledge of the domain and “specific” expertise is linked with specialized knowledge of the domain, and definitions of the levels of expertise allow us to place the business analysts in the “subexpert” group and the novices in the “layperson” group (Patel and Groen, 1991; Wineburg, 1998). Indeed, the average business analyst having a generic knowledge of IS and conceptual modeling, by their background and formation, are not as specialized as experienced modelers, but still have more expertise than novices, which are only equipped with common sense and everyday knowledge.

Thus, business analysts are used as surrogate expert modelers, by proposing for the purposes of this research that their IT competences and “generic” expertise differentiate them from novices, and thus refer to them as “experts” in the remainder of the article.

### Visual attention

The use of eye-tracking to monitor the visual attention of participants has been tested and proven as an effective way to assess the moment-to-moment cognitive processing of visual stimuli (Rayner, 1998; Bednarik and Tukiainen, 2006; Yusuf et al., 2007). Evidence suggests that attention and saccades, which are the quick movements of the eyes between different locations (Yusuf et al., 2007), are closely linked (Rayner, 1998), while fixations are linked with the cognitive processing of visual information (Just and Carpenter, 1976; Yusuf et al., 2007; Zhan et al., 2016). Technological innovations made eye-tracking instruments more accurate and reliable, while removing the need to use intrusive goggles or headset to capture precise visual data (Lupu and Ungureanu, 2013).

Multiple eye-tracking studies have evaluated the eye movement of participants during an anomaly detection task. While most of those experiments used anomalous textual sentences (Ni et al., 1998; Braze et al., 2002; Zhan et al., 2016) or anomalies in radiography (Krupinski, 2000; Reingold and Sheridan, 2011) as visual stimuli to assess the variation in eye movements, the eye-tracking methodology is quickly gaining popularity in other domains, from forensics to art (Reingold and Sheridan, 2011). These studies concluded that more fixations will land on the relevant information, which in our case is the anomalies, and that those fixations tend to become longer than the fixations on irrelevant information (Henderson and Hollingworth, 1999; Van Waes et al., 2009; Holmqvist et al., 2011).

Furthermore, the number of fixations is related to the effectiveness of the search (Goldberg and Kotval, 1999; Holmqvist et al., 2011), where a higher number of fixations usually result in an ineffective search. Finally, the total view time of the stimuli has been found to be inversely related to the detection of anomalies (Van Waes et al., 2009). Indeed, a higher time spent on a stimulus is correlated with a lower chance of identifying the anomaly; as the cognitive load increases, the probability to make errors will also increase and the general understanding of the model will decrease (Moody, 2004; Figl and Laue, 2015; Haisjackl et al., 2016).

Recent studies have used the eye-tracking methodology to investigate the understanding of process models in different comprehension tasks (Batista Duarte et al., 2021). As comprehension of process models cannot be directly observed and measured; eyetracking studies use the visual attention measures paired with comprehension correctness to identify patterns linking the scan path or cognitive load to model understanding (Petrusel and Mendling, 2013; Batista Duarte et al., 2021). As the complexity of the tasks increases (by increasing the model complexity, modeling language complexity, or reducing the participant’s knowledge of the process), the fixation duration and the total number of fixations generally increase (Petrusel et al., 2017; Zimoch et al., 2018b; Tallon et al., 2019). In general, studies conclude that analysts providing accurate answers to comprehension tasks have more and longer fixations in the relevant regions than in the irrelevant sections (Petrusel and Mendling, 2013; Zimoch et al., 2018a; Tallon et al., 2019).

These findings offer a better understanding of the visual characteristics related to successful anomaly detection in the context of conceptual modeling, which allow us to propose our first three hypotheses:

*H1 — Successful error detections in conceptual modeling will require less time spent looking at the stimulus than unsuccessful error detections.*

*H2 — Successful error detections in conceptual modeling will require, in total, fewer fixations than unsuccessful error detections.*

*H3 — Successful error detections in conceptual modeling will require, on average, shorter fixation duration than unsuccessful error detections.*

Past studies using eye-tracking in process models comprehension tasks found that visual attention and scan paths are influenced by the past work experience and personal knowledge (Petrusel et al., 2017; Zimoch et al., 2018b). However, these results are inconsistent as other research found that expertise had no immediate effect on model comprehension (Zimoch et al., 2018a; Batista Duarte et al., 2021). As the eye tracking literature is not mature enough in the field of process model comprehension, we have extended our literature review to include studies identifying the differences in visual attention between experts and novices in different types of search tasks. Those studies give us a better idea of how expertise influences visual characteristics in different domains. We keep in mind that it is not recommended to generalize eye movements meaning across tasks or domains, since contextual demands and task complexity might greatly differ (Rayner, 1998; Gegenfurtner et al., 2011).

Overall, the main findings of prior work suggest that experts tend to spend less time on a stimulus, require less and shorter fixations on average, and have all around better performance than novices (Krupinski, 2000; Gegenfurtner et al., 2011; Reingold and Sheridan, 2011; Sheridan and Reingold, 2014). These results align with our hypotheses on the visual characteristics of successful error detection, which may be explained by the tendency of experts toward more efficient search strategies and consequently better performance (Recker and Dreiling, 2007; Yusuf et al., 2007; Petrusel et al., 2017). Accordingly:

*H4 — Experts in conceptual modeling will spend less time looking at the stimulus than novices.*

*H5 — Experts in conceptual modeling will require, in total, fewer fixations than novices.*

*H6 — Experts in conceptual modeling will require, on average, shorter fixation duration than novices.*

*H7 — Experts in conceptual modeling will diagnose the anomalies more accurately than novices.*

## Materials and methods

### Participants

A within-subject experiment with one experimental factor on “error type” was conducted in order to test our hypothesis. 30 participants (15 males, 15 females, Age avg. = 28.63) were recruited and manually divided into two groups. The sample was screened to only allow participants who weren’t diagnosed with any neuropsychological conditions or major vision problems that will require glasses to use a computer, in order to meet our instrumental constraints. The research was approved by the Research Ethics Board (REB) of a large academic institution, and each participant signed a consent form and received a small monetary compensation from the university bookstore.

The “Novice” group was composed of 15 participants (9 males, 6 females) and were recruited among the volunteers enrolled in our institution’s panel. This group’s participants were between 21 and 38 years old (Avg. = 24; SD = 4.06612). Any participant with a background in IT and business analysis was excluded, leaving only those that had never used or learned any visual notation. Even though this group was mostly composed of undergraduate and graduate students, which might weaken perception of the external validity of the study, the use of students over practitioners allowed us to control the prior technique and domain knowledge of the participants in order to make sure that our novices have undeveloped IT competencies (Batra et al., 1990; Gemino and Wand, 2004; Recker and Dreiling, 2007).

The remaining fifteen (15) participants (6 males, 9 females) comprised our ‘Expert’ group. They were recruited, in part, at an International Institute of Business Analysis (IIBA) convention that took place in a major North American city in February 2018. The ages of this group range from 22 to 53 years (Avg. = 33.26; SD = 9.862161). The experts had to be business analysts and to have worked on at least 1 project using conceptual modeling, for a minimum of 15 h of work. Since each organization can develop their own “flavor” of BPMN or other notation, we also made sure to recruit experts from different institutions, in order to minimize the risk that participants will be biased by practices specific to their organization.

### Experimental stimuli

Since conceptual modeling covers such a large scale of notations and domains, we limited our choice of formalism to notations used in BPMN. The Business Process Modeling Notation, or BPMN, is an international standard for business process notation published by the Business Process Management Initiative (now Object Management Group) in 2004. BPMN strives to be understandable by all business users, from business analysts creating the models to business actors using or monitoring the processes and even developers (Birkmeier et al., 2010). Among the analytical evaluation studies, Wahl and Sindre (2006) found that BPMN is easy to learn for simple use, even though it can be more complex than other notations when used with advanced modeling concepts (Wahl and Sindre, 2006). However, empirical experiments have found no evidence that the use of BPMN over another notation would significantly improve the comprehension of the participant (Birkmeier et al., 2010; Sandkuhl and Wiebring, 2015; Jošt et al., 2016). Furthermore, the growing popularity of BPMN in the commercial and academic settings lead to its selection as the visual notation used in this experiment.

**FIGURE 1:**
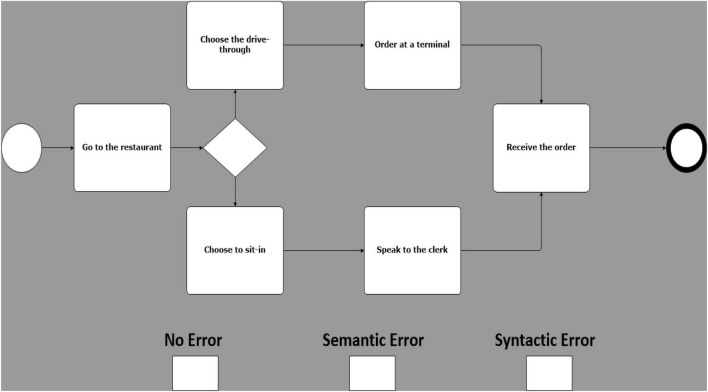
Example of a model with no error.

**FIGURE 2:**
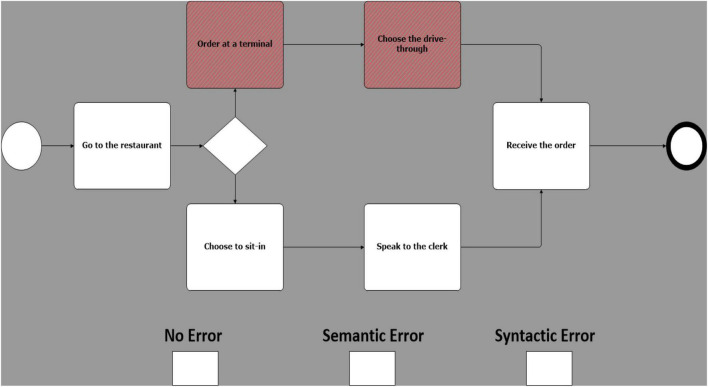
Example of a model with a semantic error (mis-ordered activities).

**FIGURE 3:**
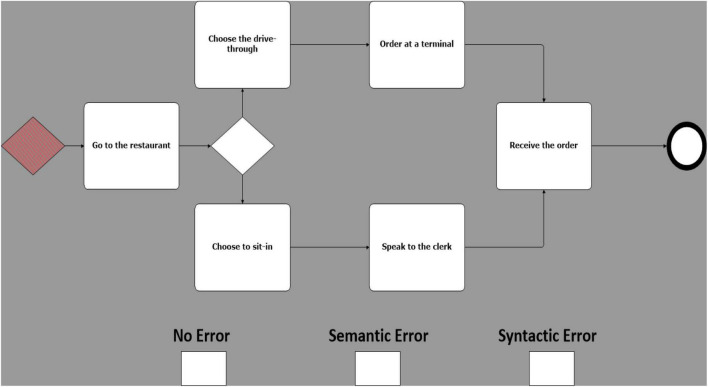
Example of a model with syntactic error (use of invalid symbols).

Building upon the experimental design proposed by Davis et al. (2018), 25 models were created, equally grouped in 5 different business scenarios. Each of these models was then duplicated in 3 copies called sentences. Sentences were then manipulated on a single experimental factor, error type. There are 3 error types: one with no known errors (Figure 1); one with a known semantic error (Figure 2), and one with a known syntactic error (Figure 3) (Davis et al., 2018). Therefore, 75 models, 25 models for each error type, were created (i.e., 25 with no errors, 25 with sematic errors and 25 with syntactic errors). Boxes indicating the three error types were added at the bottom of each model and were used by participants to indicate their diagnosis.

Syntactic errors include the use of invalid symbols (Davis et al., 2018) (e.g., the use of a BPMN “start event” symbol to represent a “gateway”) or a non-consistent flow (e.g., a misdirected flow between two activities). Semantic errors, on the other hand, are subtler and cannot be identified at a glance, in addition to being difficult to recognize by a compiler or other verification technologies, making them especially costly and hard to correct (Dijkman et al., 2008). By using valid symbols but ambiguous design (e.g., sequence of activities in a scenario mis-ordered), they present an unintended and puzzling message.

While several studies found that the domain knowledge had no significant effect on the understanding of the models in a model comprehension task (Recker and Dreiling, 2007; Bera, 2012; Recker et al., 2014; Turetken et al., 2016), we feared that the disparity in the prior knowledge of the business domains of the models between the participants would greatly influence the ease with which they would pinpoint errors in an anomaly detection task (Gemino and Wand, 2004; Birkmeier et al., 2010). Therefore, we based our models on simple and well-known scenarios to all participants (e.g., fast food ordering process). By limiting the range and quantity of symbols used between 8 and 13 elements per model, we controlled the complexity of each scenario and models (Zur Muehlen and Recker, 2008; Sánchez-González et al., 2010). In accordance with prior BPMN literature (Wahl and Sindre, 2006), only the basic symbols were used, since the use of more advanced components of the notation greatly complicates the comprehensibility of the models. Exclusive gateways were used, since the use of gateways has a positive effect on the comprehension of a model (Recker, 2013), but we did not use any of the other types of gateway since a use of a heterogeneous range of gateways tends to lower the comprehension of the models (Sánchez-González et al., 2010). By combining the use of readily comprehensible models with a training presentation and some practice tasks, we partly mitigated the effect of the variation of prior technical knowledge between each participant (Bavota et al., 2011).

The training consisted of a PowerPoint presentation explaining the symbols used and the two types of errors present in the experiment (i.e., semantic and syntactic errors). The participants went through the presentation at the beginning of the experiment, after the calibration of the instruments. They were allowed to take as much time as needed. The content of the presentation was tested on 6 participants with no, or close to no, experience with BPMN. After reading the presentation, the pretested participants were asked to describe the different symbols and rules explained in the training. The training stimuli were then improved, and any confusion removed.

After the training presentation, the participants were given a practice exercise. Just like the experiment, the practice consisted of identifying and diagnosing an error in a conceptual model. The task was composed of three (3) models, where each one had a different type of error (i.e., no error, semantic error, and syntactic error). To avoid any form of bias, the practice models were not related to the scenarios used later in the experiment and the modeling (experimental) environment was the same (Bavota et al., 2011). The only difference between the training task and the experiment was that the participant could see the correct answer after each practice model. This way, any remaining confusion regarding the error types was ameliorated before the experiment.

The training presentation and the practice task allowed us to make sure every participant had the necessary knowledge to complete the experiment and to mitigate the effect of learning through trial (Bavota et al., 2011). While useful with novices and experts who were less familiar with BPMN, the training phase of the experiment also allowed us to make sure that the experts accustomed with the use of BPMN were still using the normalized rules of BPMN and were not biased by some of their own organization’s standards.

**FIGURE 4:**
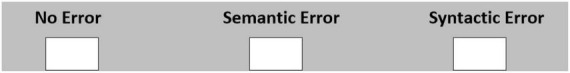
Boxes indicating error types.

To complete the tasks, the participant had to manually click on the modeling error and on the box classifying the error type at the bottom of the screen, using the mouse (see Figure 4). After each click on the model, a visual indicator would be placed on the location of the click and would disappear after 0.5 s. This indicator was used to provide visual feedback to the participant that the click was registered, in order to mitigate any confusion about the user’s actions. If the model contained no error, the participant simply had to click on the box indicating “No Error.” After identifying the error, by clicking on it, and the error type, by clicking on the corresponding box, participants manually advanced to the next model by pressing the spacebar. After completion of a scenario, the researcher opened and completed an online questionnaire. The researcher then closed the questionnaire and started the next set of models, or ‘scenario’.

### Protocol

As the participants arrived at the laboratory lobby, the researcher greeted them and explained roughly the stages of the experiment. They were then asked to read and sign the consent form, while the experimenter made sure that the equipment was ready to run and that all the required software was initiated. After the consent form was signed by both the participant and the experimenter, participants were taken to the laboratory and the eye-tracking device calibrated. These steps took approximately 10 min.

**FIGURE 5:**
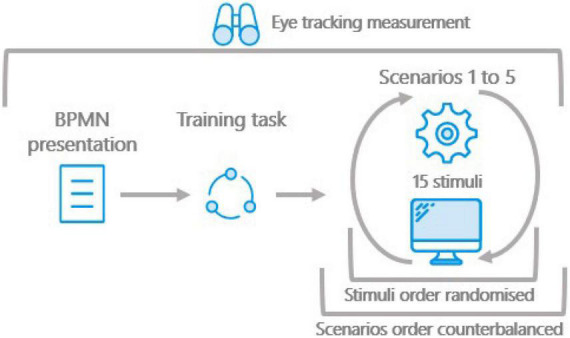
Experimental design.

The participant then went through the training presentation and practice task, which took, on average, between 5 and 7 min to complete. On completion of the practice task, the participants started their first task for a random scenario. For each scenario, the participants had to identify and diagnose errors in 15 models, shown in a random order, without any time limitations. After completing a scenario, the participants would then start another error detection task, for another scenario at random. The experiment would conclude when a participant would go through the 5 scenarios, totaling the 75 models, and a questionnaire on their previous experience with conceptual modeling. At the end of the experiment, which took around 45 min, the participant, was given their compensation and escorted back to the building lobby. Figure 5 summarizes the experimental protocol design.

### Measures

The experiment was set up using SMI Experiment Center 3.7.56 and the data were processed and analyzed using SMI BeGaze 3.7.40 software. The stimuli were created using Microsoft Visio 2010. The statistical analysis was carried out using Stata/MP 15.1. We captured the behavioral measures, which translate into eye movements, using SMI RED 250 eye-tracker (Red 250, SensoMotoric Instruments GmbH, Teltow, Germany). The instrument was configured at a sampling frequency of 60 Hz and a fixation duration threshold of 200 ms (Rayner, 1998; Holmqvist et al., 2011). Following the calibration, using a 9-point predefined calibration grid, the eye-tracker was adjusted for each participant, to a gaze-position deviation of 0.5° or less.

**FIGURE 6:**
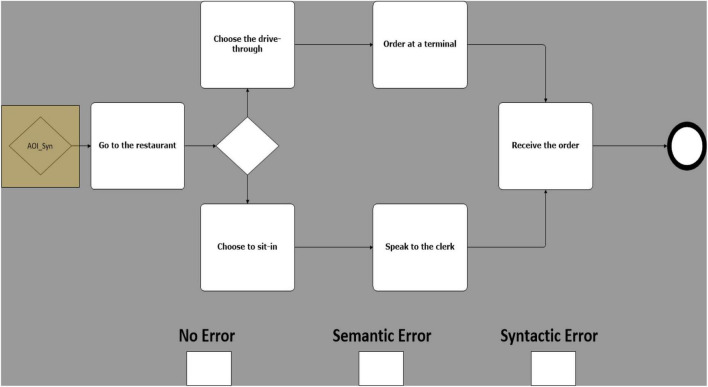
Example of a model with visible AOI.

For each model, areas of interest (AOIs) were mapped to the location of the error. Additional margins of at least 1.5° were added to the AOIs to mitigate the imprecision of the eye-tracker (Holmqvist et al., 2011). Figure 6 shows an example of a model with AOIs, where the AOI can be seen on the syntactic error. These AOI mappings allowed us to gather data on the proportion of fixation and time to first fixation on precise locations in our models.

As the literature tends to agree that the cognitive processing of visual stimuli is done during fixations (i.e., the stabilization of the eye on an object) (Just and Carpenter, 1976; Yusuf et al., 2007; Zhan et al., 2016), we gathered the fixations, and their duration, inside each area of interest (AOI). We also took into account the total view time of each stimulus, in order to evaluate the response time of the participants.

The error rate for each participant was also determined. A performance score was created for the three types of error and calculated by manually reviewing, with the help of the experiment’s recordings, each answer given by the participants. Those scores, in percentage, were used to identify which type of error was the hardest to diagnose and for which error group the difference between experts and novices was the largest.

A questionnaire was created in order to determine the prior experience of the participants. It was composed of 7 questions assessing the number and kinds of visual notations known by the participant, the number of projects and hours spent working on conceptual models and the kind of manipulation done in those projects. In accordance with literature on expertise, rather than using the amount of time spent as a business analyst as our indicator of modeling expertise, since experience in itself is often a poor predictor of true expertise (Gobet, 2016), we chose objective measures of modeling experience, being the number of projects involving conceptual models in which the participant took part and the hours spent working with conceptual models, in BPMN or any other notations, to define our experts and novices.

### Analysis

To test our hypotheses, linear regressions with mixed model and a two-tailed level of significance were performed. We have used a generalized linear mixed model using proc glimmix in SAS SAS9.4. For this type of presentation study, using a generalized linear mixed model is more flexible than repeated ANOVA as it can take into account control variables (Judd et al., 2017; Plonsky and Oswald, 2017). The repeated measures ANOVA results based on type 3 sum of squares calculated using Proc Glimmix in SAS are shown in the Appendix. Dummy variables were created to represent the error types: dErrSem for semantic errors, dErrSyn for syntactic errors with invalid symbols (ErrSyn2) and with non-consistent flow (ErrSyn3), dErrSyn2 only for syntactic errors with invalid symbols and dNoError for stimuli with no error. Syntactic errors with non-consistent flow (ErrSyn3) are isolated when dErrSyn = 1 and dErrSyn2 = 0 and, therefore, no dummy variable dErrSyn3 variable was created. The binary variable Expertise was also created to distinguish our two groups of participants, where Expertise = 1 when the participant is a business analyst. The variable dWhiteSpace regroups everything that is not inside an AOI and is considered as ‘irrelevant’ information. For each stimulus with an error (thus excluding stimuli with dNoError), there will be a dummy variable (dErrSem, dErrSyn or dErrSyn2) representing the error area and dWhiteSpace representing the rest of the stimulus. Scenarios and sentences were not included in the analysis as they were presented in a counterbalanced way, and, based on our pretest, they were considered of the same difficulty level, length, and complexity. Also, we had no hypothesis on effect of the scenarios nor sentences.

By creating a median split on the overall performance of experts, we can create and compare two groups: the performing and underperforming experts. This manipulation allows us to push our analysis further, and to articulate the heuristics of experts with good and poor performance. The dummy variable “dGoodExpert” was then created, where dGoodExpert = 1 represents the group of high performing experts. A similar manipulation was carried out by creating a median split on the performance of all participants, thus creating dPerformance, where dPerformance = 1 represents the group of high performing participants, novices and experts alike. The results of the linear regressions and correlation are shown in the tables in the following section.

## Results

Tables 1A,B present the results of our experiments. The first hypothesis (H1) states that successful identification and diagnosis of errors in conceptual models will take less time than unsuccessful answers. We compared the effect of the variable “Answer” (i.e., if the participant successfully diagnosed the error, Answer = 1, if the diagnostic was wrong, Answer = 0) on the measure “Total View Time.” A significant relationship between those two variables was found (*b* = –0.4143, *p* < 0.001), meaning that correct diagnostics tend to be looked at for a significantly shorter amount of time than wrong answers, thus supporting the hypothesis. Furthermore, Table 1B shows that syntactic errors tend to be diagnosed faster than other error types (*b* = –0.3237, *p* < 0.001) and, more specifically, stimuli with syntactic errors that used invalid symbols (ErrSyn2) are diagnosed faster than stimuli with non-consistent flow (ErrSyn3) (*b* = –0.3258, *p* < 0.001). These results contrast with those for the ‘No Error’ group, which were looked at longer than other error types (*b* = 0.2913, *p* < 0.001). No statistically significant result was found for semantic errors.

**TABLE 1A:** Results of linear regressions.

	Sex	LogAge	Expertise	dPerformance	dGoodExpert
Perf_Total	–0.0674 (0.0380)	–0.0911 (0.0807)	0.0052 (0.0389)	0.1193** (0.0352)	0.0941 (0.0491)
Total view time	0.2373** (0.0724)	0.3843* (0.1553)	0.0831 (0.0821)	–0.0648 (0.0836)	0.0834 (0.1351)
Time to first fixation	0.0599 (0.117)	–0.1358 (0.2955)	–0.0247 (0.1170)	0.1912 (0.0463)	0.1855 (0.1782)
Fixation count	0.1664* (00629)	0.2220 (0.1380)	0.1168 (0.0681)	0.0152 (0.0463)	0.1005 (0.1191)
Fixation duration (ms)	10.9187** (3.2504)	15.5805* (67133)	6.2263 (3.6720)	–2.3073 (3.7416)	2.5698 (6.2372)
Fixation duration (%)	–0.0423 (0.0524)	–0.1289 (0.1151)	–0.0040 (0.0530)	0.0298 (0.0539)	0.0159 (0.0948)
Perf_NoError	–0.1276** (0.0446)	–0.1876 (0.1134)	–0.092 (0.0470)	0.1483** (0.0476)	0.1921* (0.0655)
Perf_Sem	0.0025 (0.0478)	0.0455 (0.0835)	0.0415 (.0462)	0.0962* (0.0470)	0.0110 (0.0540)
Perf_Syn	–0.0771 (0.0647)	–0.1309 (0.1407)	0.0680 (0.0633)	0.1134 (0.0626)	0.077 (0.0815)
Perf_Syn2	0.0470 (0.0769)	–0.0909 (0.1795)	0.0330 (0.0776)	0.0453 (0.0792)	–0.0084 (0.1163)
Perf_Syn3	–0.1601* (0.0681)	–0.1571 (0.1546)	0.0912 (0.0695)	0.1605* (0.0680)	0.1409 (0.0883)
Answer	–0.3469 (.1974)	–0.4304 (0.3991)	0.0417 (0.2061)	0.6172*** (0.1804)	0.4944* (0.2395)
Input	0.5618 (0.348)	0.5629 (0.6923)	0.6165 (0.3288)	–0.5169 (0.3470)	–0.9732* (0.4033)

Standard errors in parentheses; signif.: ***p < 0.001, **p < 0.01, *p < 0.05.

**TABLE 1B:** Results of linear regressions.

	dNoError	dErrSem	dErrSyn	dErrSyn2	Answer
Perf_Total	0.0001 (0.0001)	–0.0001 (0.0001)	0.0001 (0.0001)	–0.0014 (0.001)	0.0602*** (0.0128)
Total view time	0.2913*** (0.0404)	0.0325 (0.0316)	–0.3237*** (0.0460)	–0.258*** (0.0501)	–0.4143*** (0.0314)
Time to first fixation	–3.7968*** (0.1215)	1.8252*** (0.0785)	2.0672*** (0.0754)	–0.192 (0.1082)	–0.2053 (0.1546)
Fixation count	2.2335*** (0.0473)	–0.7364*** (0.0295)	–1.4956*** (0.405)	–0.2820*** (0.0520)	0.1673 (0.0942)
Fixation duration (ms)	1.02e + 02*** (3.6638)	–4.09e + 01*** (1.8398)	–6.09e + 0*** (2.3814)	–7.8289*** (1.5880)	1.6253 (4.4275)
Fixation duration (%)	1.9532*** (0.0410)	–0.7196** (0.0336)	–1.2322*** (0.0612)	–0.0044 (0.0663)	0.6760*** (0.0922)
Perf_NoError	–	–	–	–	–
Perf_Sem	–	–	–	–	–
Perf_Syn	–	–	–	–	–
Perf_Syn2	–	–	–	–	–
Perf_Syn3	–	–	–	–	–
Answer	0.2365 (0.1658)	–0.4336** (0.357)	0.2191 (0.1685)	0.0871 (0.2088)	–
Input	4.3134*** (0.7370)	–1.4103*** (0.2027)	–0.4654* (0.1923)	–1.1883*** (0.344)	–

Standard errors in parentheses; signif.: ***p < 0.001, **p < 0.01, *p < 0.05.

**FIGURE 7:**
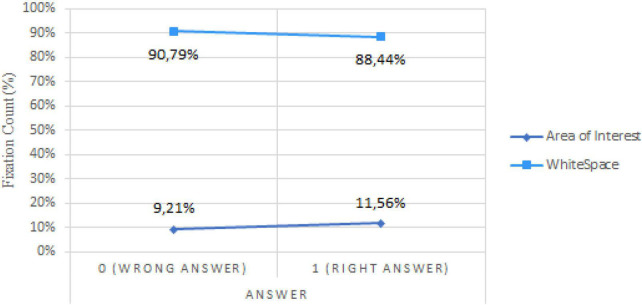
Fixation count by AOI and answer.

While H2 states that successful error detection will require fewer fixations than incorrect diagnostics, no significant results substantiate this assumption. To the contrary, a trend in our sample suggests that “Answer” seems to have a positive relationship, but insignificant, with “Fixation Count” (*b* = 0.1673, *p* < 0.0864), implying that good answers could be linked with a higher fixation count. However, while the fixation count in the AOI is lower than in the White Space (i.e., everything that is not inside the AOI) for both correct and incorrect diagnostic, the proportion of fixation in the relevant area is higher for the accurate diagnostics of syntactic errors (*b* = 0.0236, *p* < 0.003) as we can see in Figure 7.

Similar results were found for semantic errors (*b* = 0.0062, *p* < 0.022) and syntactic errors of non-consistent flow (*b* = 0.0196, *p* < 0.008).

No statistically significant results were observed for H3, which states that successful error diagnosis will be linked with shorter fixation duration. H4, which states that experts in conceptual modeling will spend less time looking at the stimulus than novices, is also not supported since no statistically significant results were found linking “Expertise” with “Total View Time.” However, Table 2, which combines statistical analysis of a dataset when only including the experts (Expertise = 1), suggests that among experts, syntactic errors tend to be diagnosed faster than other error types (*b* = –0.4167, *p* < 0.001), with the “invalid symbols” group being diagnosed faster than the ‘non-consistent flow’ errors (*b* = –0.3189, *p* < 0.01), and where stimuli without any errors were diagnosed slower (*b* = 0.3716, *p* < 0.0038). These results concur with the findings for H1. Indeed, just like accurate diagnostics, experts tend to identify syntactic errors faster and diagnose error-free stimuli slower. Again, no statistically significant result was found for semantic errors.

**TABLE 2:** Effect of errors type on attentional characteristics, when Expertise = 1.

	dWhiteSpace	dErrSem	dErrSyn	dErrSyn2
Total view time	0.3716** (0.0455)	0.0457 (0.0423)	–0.4167*** (0.0652)	–0.3189** (0.0921)
Fixation count	1.8606*** (0.0629)	–1.0475*** (0.0499)	–1.7427*** (0.0681)	–0.3491*** (0.0606)
Fixation duration (ms)	80.8083*** (4.3135)	–5.12e + 01*** (3.1097)	–7.00e + 01*** (3.9850)	–1.04e + 01*** (1.6075)
Fixation duration (%)	1.7238*** (0.0811)	–1.0859*** (0.0440)	–1.4991*** (0.1128)	–0.1308 (0.0770)

Standard errors in parentheses; signif.: ***p < 0.001, **p < 0.01.

Table 3 shows the significant results from statistical analysis from a dataset only including the high performing experts (dGoodExpert = 1). A similar pattern can be found, where, among these proficient experts, syntactic errors are diagnosed faster than other types of errors (*b* = –0.4884, *p* < 0.0013), with faster identification of errors involving invalid symbols usage rather than non-consistent flow (*b* = –0.3489, *p* < 0.008) and a longer total view time with stimuli in the “No Error” group (*b* = 0.3683, *p* < 0.0005). However, a statistically significant link was found for the semantic errors, where the high performing experts tend to respond more slowly to stimuli with semantic errors than other error types.

**TABLE 3:** Effect of errors type on attentional characteristics, when dGoodExpert = 1.

	dWhiteSpace	dErrSem	dErrSyn	dErrSyn2
Total view time	0.3683*** (0.0622)	0.1201 (0.0607)	–0.4884** (0.0955)	–0.3489** (0.0953)
Fixation count	1.8245*** (0.0629)	–1.0295*** (0.0830)	–1.7074*** (0.0861)	–0.4257** (0.0847)
Fixation duration (ms)	80.3340*** (5.3879)	–5.01e + 01*** (4.7160)	–7.04e + 01*** (4.3249)	–1.23e + 01** (2.5090)
Fixation duration (%)	1.6751*** (0.1096)	–1.1033*** (0.0773)	–1.4095*** (0.1364)	–0.1725 (0.0944)

Standard errors in parentheses; signif.: ***p < 0.001, **p < 0.01.

H5 and H6 were found to be inconclusive since no statistically significant result was observed. The effect of “Expertise” on “Fixation Count” (*b* = 0.1168, *p* < 0.0970) and “Fixation Duration” (*b* = 6.2263, *p* < 0.1007).

H7, which proposes that experts should diagnose anomalies more accurately than novices, was tested by analyzing the effect of “Expertise” on the “Total Performance” and the individual performance for each error type (see Table 1A). While no significant values were found, a trend found in our sample for the performance with the “No Error” group of stimuli, shows that, contrary to our expectations and hypothesis, experts tend to have a lower performance for error-free stimuli than novices (*b* = –0.0942, *p* < 0.0547).

To get a deeper understanding of the relationship between our classification of expertise and the responses to the stimuli, we analyzed the effect “Expertise” on the variable “Input” for wrong diagnostics, where Input = 1 means that the participant diagnosed a wrong anomaly and Input = 0 denotes that the participant wrongly thought that the stimuli didn’t have any error. While not significant, the trend in our data suggests that experts tend to diagnose wrong anomalies more than novices (*b* = 0.6165, *p* < 0.0608). These false (or secondary) positives were unexpected. Like error free code, completely unambiguous BPMN models are a utopian myth. Although clearly a limitation, the paradox evident here prompts important future research questions about the soundness of the expert-novice dichotomy.

Contrary to our expectations, substituting “Expertise” with “dGoodExpert” showed that high performing experts didn’t find errors more frequently than underperforming experts (*b* = –0.9731, *p* < 0.0158), suggesting that they diagnose more “false negatives.” However, high performing experts are also associated with a higher success rate with error-free stimuli than other participants (*b* = 0.1921, *p* < 0.0109). These confounding results are further highlighted when we isolate the error types of the stimuli wrongly diagnosed. We can see in Table 4 that experts, when offering a false diagnosis, tend to identify false anomalies in syntactic errors with invalid symbols use (*b* = 1.2481, *p* < 0.0048) more than novices. Despite this finding, the effect of “Expertise” on “Input” for semantic errors in our sample was positive, but statistically insignificant (*b* = 0.2152, *p* < 0.5858), while high performing experts are more inclined to inappropriately respond “No Error” than underperforming experts (*b* = –1.1518, *p* < 0.0187).

**TABLE 4:** Effect of expertise on the type of error answered, when Answer = 0.

	Expertise	dGoodExpert
Input	0.6165 (0.3288)	–0.9733* (0.0158)
Input (Error type = 1)	0.2152 (0.3948)	–1.1518* (0.4895)
Input (Error type = 2)	1.2481** (0.4425)	0.6205 (0.5549)
Input (Error type = 3)	0.958 (0.5517)	–0.7816 (1.0133)

Standard errors in parentheses; signif.: **p < 0.01, * *p* < 0.05.

The ANOVA analyses provided in the Appendix confirm the results from the linear regressions used in Tables 1–4. All ANOVAs are repeated measures ANOVA based on type 3 sum of squares after controlling by the order of stimulus shown to the participants, except for the performance measures as they are not repeated measures and are not affected by the stimulus order. The performance measures include the following measures: Perf_Total, Perf_NoError, Perf_Sem, Perf_Syn, Perf_Syn2, and Perf_Syn3.

Table 5 presents correlations between the participants’ answers of the questionnaire on previous experience with conceptual modeling and their performance in the experiment. Contrary to our expectations, our analysis shows that, prior experience is negatively correlated with performance. For instance, we observe a non-significant trend in our sample where Nb_Q2, which is the number of notations previously experienced, has a negative effect on performance (*b* = –0.3410, *p* < 0.0703). This infers – again, paradoxically, that participants with experience of more notations beforehand tend to have a lower score. Similarly, lQ3_cont, which represent the number of projects in which they used any visual notation, has a negative impact on performance (*b* = –0.3991, *p* < 0.0320).

**TABLE 5:** Correlation table of items of Past Experience questionnaire.

	dQ2	Nb_Q2	lQ3_cont	lQ4_cont	lQ6_cont	lQ7_cont
Performance	–0.2183	–0.3410	–0.3991*	–0.2828	–0.3294	–0.2245
dGoodExpert	0.2774	–0.2206	–0.3480	–0.2122	–0.0255	0.1168

Signif.: *p < 0.05.

Table 6 summarizes our analyses of the data and the results of hypothesis testing.

**TABLE 6:** Summary of hypotheses.

Hypothesis	Description	Conclusion
H1	Successful error detections in conceptual modeling will require less time spent looking at the stimulus than unsuccessful error detections.	Supported
H2	Successful error detections in conceptual modeling will require, in total, fewer fixations than unsuccessful error detections.	Not supported
H3	Successful error detections in conceptual modeling will require, on average, shorter fixation duration than unsuccessful error detections.	Not supported
H4	Experts in conceptual modeling will spend less time looking at the stimulus than novices.	Not supported
H5	Experts in conceptual modeling will require, in total, fewer fixations than novices.	Not supported
H6	Experts in conceptual modeling will require, on average, shorter fixation duration than novices.	Not supported
H7	Experts in conceptual modeling will diagnose the anomalies more accurately than novices.	Not supported

## Discussion

Clarifying the nature of expertise in conceptual modeling is crucial to improve business analysts’ training curriculum. Even if the characteristics of visual attention related to optimized searches within visual stimuli are known, expertise can translate into several behavioral dimensions that – as our results suggest – may be orthogonal, depending on the domain. Expert radiologists can identify an anomaly more quickly in a visual stimulus (Krupinski, 2000), while an expert probation officer will tend to take more time than a novice during a file reconstruction exercise (Lurigio and Carroll, 1985).

Thus, by comparing the performance, the visual attentional characteristics and the antecedents between business analysts and novices in conceptual modeling, our main objective is to deepen insight into the interdependent heuristics of experts and how dimensions of expertise affect the behavior and performance of the participants.

First, we compare the attentional characteristics of correct and incorrect diagnosis, in order to compare our results with prior experiments. While H1 was supported, indicating that correct diagnostics tend to take a shorter amount of time than incorrect answers, which concur with prior findings among the literature (Van Waes et al., 2009), H2 and H3 were not. Contrary to what has been observed in prior work, the accurate diagnostics in this experiment weren’t linked with a shorter amount of fixation count or fixation duration (Henderson and Hollingworth, 1999; Van Waes et al., 2009; Holmqvist et al., 2011). While not statistically significant, the results from our sample contradict previous findings and are interesting, since they point toward an increase rather than a decrease in fixation count and duration. This might be explained by the complexity of the task. Indeed, it has been postulated that a lower amount of fixation could mean that the task was merely too simple, therefore necessitating a lesser amount of cognitive processing (Gegenfurtner et al., 2011). In contrast, the fact that the task may require a larger amount of fixation and fixation duration on a stimulus to be successfully completed may show that the anomaly detection process in conceptual modeling is more cognitively complex than similar tasks in other domains. This postulation is supported by known ambiguity of conceptual models and the challenge they present in terms of comprehension (Figl, 2017). It therefore becomes clear that a certain amount of fixation is needed to fully understand the models and that trials with a lesser amount of fixation count and duration are linked with incorrect answers, since the participants may have under-appreciated the conceptual richness of the stimulus, leading to premature and inaccurate diagnosis.

Second, by comparing the error detection process between the different error types, we aim to better understand the relation between model comprehension and the semantic and syntactic dimensions of conceptual modeling. Syntactic errors involving invalid symbols use is the type of error diagnosed the fastest, leading us to surmise that they may be the easiest kind of error to spot. This concurs with prior work on syntactic and semantic errors, where syntactic errors are found to be less subtle and easier to recognize than semantic errors, leading to a lesser amount of time needed to be diagnosed (Dijkman et al., 2008; Davis et al., 2018).

An interesting finding arises from the comparison of the two kinds of syntactic errors. Stimuli with seeded syntactic errors of non-consistent flow tend to be viewed for a longer amount of time than models with syntactic errors that used invalid symbols, thus suggesting greater complexity. While more tests and analysis are needed to better understand the full nature of this complexity, we are aware of no study that compared those two kinds of syntactic errors, and, therefore, we believe it is a lead worth investigating further. Contrary to expectations, stimuli with no seed errors were answered slower than other error types. This can be explained by the concept of ‘stopping rule’, which is the extent to which participants would continue or terminate their search for additional information before taking a decision (Nickles et al., 1995; Browne and Pitts, 2004). It is then reasonable to expect that the participant takes more time to diagnose that a model has no errors than the average time needed to diagnose a semantic or syntactic error, since they must pass through the same cognitive process, without stopping their search at the first error found.

The most interesting – and surprising – results appear when we compare experts with novices. Contrary to our hypotheses and prior studies, we found that experts were not more efficient and effective than novices in our experimental tasks. We found that experts did not have fewer fixations, fixation duration or total view time than novices, and even had lower performance than novices for error-free stimuli. How to explain this confounding result?

While some studies have found that experts may behave similarly to novices, especially in tasks requiring judgment (Goldberg, 1959; Levy and Ulman, 1967; Bédard, 1989), few experiments have produced results where novices performed better than experts (Adelson, 1984; Köpke and Nespoulous, 2006). In these experiments, the qualitative difference between how experts and novices perform the tasks would influence which task was more suited for novices and which for experts. The task type and complexity would then influence which group had a better performance. For example, Adelson (1984) found out that the type of representations constructed by computer programmers was different between novices and experts, and that each type of representation was more suited for a specific type of task.

This leads us to propose that our task type, or the level of complexity of the task, may have been more suited to novices: this might partly explain the unexpected results. Conceptual models are – by their very name and nature – potent with ambiguities that business analysts must process cognitively to find anomalies and errors. However, the use of simple models, as used in this experiment, could lead the experts to over-complexify the stimuli when trying to find all the ambiguities. Our finding that experts tend to diagnose more non-existent anomalies than novices, could lead to more false diagnoses, more time spent on the stimuli and more fixations. In other words, experts tend to *over think* anomaly detection on simple conceptual models. They expect to find more defects than do novice subjects.

Biases linked to expertise also need to be taken into consideration, since cognitive bias is considered one of the most serious handicaps of experts (Ericsson et al., 2006). Studies in neurophysiology have found that experts tend to activate the areas of the brain associated with inhibition more than novices, suggesting that experts must inhibit misconceptions in order to give a sound answer (Masson, 2007; Masson et al., 2014; Brault Foisy et al., 2015). Furthermore, experts tend to have more design fixations, such as functional fixedness (i.e., restricting the use of an object to previously encountered functions) or mental sets, which limit the creativeness and set of ideas used in problems solving (Jansson and Smith, 1991; Ericsson et al., 2006). In our situation, this bias is extremely important, since understanding and diagnosing unknown models, without any context or clues, requires a fair amount of creativity and cognitive flexibility. Cognitive schemata arise from prior experiences. They frame and, in some ways, limit their abilities to inhibit misconceptions. The creative limitations from this bias could very well explain why most experts tend to look extensively for an error and find ambiguities even in error-free stimuli. This claim is supported by the *post hoc* analysis showing that high performing experts seem to manage to overcome this bias and successfully diagnose error-free stimuli. Clearly, further studies and experiments are warranted to explore the deeper insights indicated by the contradictory results.

Our main theoretical and practical contribution lay in the elaboration of the expert-novice dichotomy in conceptual modeling. Our findings regarding experts’ performance, and the unexpectedly narrow difference between that of experts and novices, reveals a paradox that offers a new perspective on the richness of the cognitive *milieu* of expertise in modeling. From a theoretical perspective, our results highlight a clear need for more in-depth studies on how business analysts process and comprehend conceptual models compared to novices. At a practical level, articulating the difference between semantic and syntactic errors, and the difference between the two kinds of syntactic errors, will enable instructors to adapt and improve their curriculum when training new business analysts. Demonstration that different error types require different levels of cognitive processing, and that experts may over-think and over-complexify their representation of a model when looking for anomalies in error-free stimuli, are important to take in consideration when trying to develop novices into experts.

### Limitations and future research

This paper presents an initial attempt to articulate the differences between experts and novices in an anomaly detection task in conceptual modeling: the cognitive complexity of both the field and the study give rise to limitations and opportunities for improvement in future work.

While we use the concept of IT competence (Bassellier et al., 2003) to propose a link between experience as a business analyst and expertise in conceptual modeling, we do not actually control or measure the level of IT competence of our participants. Rather than using a conceptual model based on the model of Bassellier et al. (2003) to measure the antecedents of IT competence, we used years of experience as a business analyst as a surrogate indicator of competence. An interesting way to extend this experiment would be to study the actual independent variables of IT competence that would contribute to improving the business analysts’ expertise in conceptual modeling. Furthermore, it would be interesting to widen the range or scope of expertise, by comparing participants in 3 or 4 groups with different levels of experience. This would allow us to further explore the orthogonality of the characteristics and “dimensions” of expertise, rather than trying to compare what we now primarily see as a binary concept with two samples. Future research should consider using a knowledge test to qualify the expertise of the business analysts. This could allow capturing with more richness the effect of the level of expertise.

A further limitation of our study is the fact that we didn’t control for domain expertise (or semantic expertise), and rather use simple and well-known scenarios. By collecting data on the familiarity of participants for each scenario, we may have a better insight into what really causes such small difference between novices and experts. We only manipulated the “error type” factor and did not manipulate the complexity of the models. In the real world, models can sometimes be more complex with 50 activities and more, and our study relied on process models with a number between 8 and 13 activities. Future research should explore the effect of the domain of expertise and level of complexity as they are likely to play a role in the ability of the analyst to detect errors as seen in the study by Haisjackl et al. (2018).

New measures, such as analyzing and comparing the scan paths and stopping rules of novices and experts, have the potential to offer us valuable information on reading techniques used by experienced modelers which could, in turn, be used to provide recommendations for teaching curricula, the design of instructional materials and the revision of standardized notations such as BPMN.

Since the acquisition of skill can bring major changes to the brain activity and areas activated, depending on the skill and the training, a study using functional magnetic resonance imaging (fMRI) techniques or EEG might allow us to pinpoint the domain-general control areas and the domain-specific representational areas associated to expertise in error detection tasks during conceptual modeling. Considering that the performance of those areas is sensitive to the nature of the training, by identifying which areas of the brain to work on, we could create training curricula and materials strengthening those areas, thus improving the cognitive processing of future business analysts. Furthermore, a more detailed understanding of the cognitive processing of experts will grant us valuable insights into how novices and experts differ, overcoming the limitations of the apparently false dichotomy that currently persists in the literature.

## Data availability statement

The datasets presented in this study can be found in online repositories. The names of the repository/repositories and accession links provided upon request from the authors.

## Ethics statement

The study was reviewed and approved by the Ethics Committee of HEC Montreal (Certificate #2018-3047, March 3rd 2018). It was conducted according to the guidelines of the Declaration of Helsinki. The patients/participants provided their written informed consent to participate in this study.

## Author contributions

All authors listed have made a substantial, direct, and intellectual contribution to the work, and approved it for publication.
