# Supplementary material for: Don’t overthink it: The paradoxical nature of expertise for the detection of errors in conceptual business process models
Source: Front Neurosci. 2022 Nov 24;16:982764. doi: 10.3389/fnins.2022.982764 (PMC9731113; doi:10.3389/fnins.2022.982764)
Supplement: Supplementary file 1 [file Data_Sheet_1.docx]

**Appendix – ANOVA Statistics for Tables**

**ANOVA Statistics for Table 1.A. and Table 1.B.**

| Dependent Variable | Effect | NumDF | DenDF | FValue | ProbF |
| --- | --- | --- | --- | --- | --- |
| Total view Time | Sex | 1 | 2128 | 12.72 | 0.0004 |
| Total view Time | LogAge | 1 | 2127 | 9.06 | 0.0026 |
| Total view Time | Expertise | 1 | 2128 | 1.7 | 0.1931 |
| Total view Time | dPerformance | 1 | 2128 | 0.83 | 0.3616 |
| Total view Time | dGoodExpert | 1 | 1018 | 0.14 | 0.7092 |
| Total view Time | dNoError | 1 | 2127 | 53.88 | <.0001 |
| Total view Time | dErrSem | 1 | 2127 | 1.1 | 0.2941 |
| Total view Time | dErrSyn | 1 | 2127 | 51.14 | <.0001 |
| Total view Time | dErrSyn2 | 1 | 688 | 44.99 | <.0001 |
| Total view Time | Answer | 1 | 2127 | 152.48 | <.0001 |
| Time to first fixation | Sex | 1 | 2062 | 0.29 | 0.5889 |
| Time to first fixation | LogAge | 1 | 2061 | 0.19 | 0.6654 |
| Time to first fixation | Expertise | 1 | 2062 | 0.03 | 0.8566 |
| Time to first fixation | dPerformance | 1 | 2062 | 2.44 | 0.1184 |
| Time to first fixation | dGoodExpert | 1 | 981 | 1.14 | 0.2852 |
| Time to first fixation | dNoError | 1 | 2061 | 1001.14 | <.0001 |
| Time to first fixation | dErrSem | 1 | 2061 | 562.87 | <.0001 |
| Time to first fixation | dErrSyn | 1 | 2061 | 766.38 | <.0001 |
| Time to first fixation | dErrSyn2 | 1 | 625 | 3.34 | 0.0683 |
| Time to first fixation | Answer | 1 | 2061 | 1.87 | 0.1721 |
| Fixation count | Sex | 1 | 2128 | 7.18 | 0.0074 |
| Fixation count | LogAge | 1 | 2127 | 3.15 | 0.0759 |
| Fixation count | Expertise | 1 | 2128 | 3.38 | 0.0662 |
| Fixation count | dPerformance | 1 | 2128 | 0.02 | 0.8882 |
| Fixation count | dGoodExpert | 1 | 1018 | 0.57 | 0.4504 |
| Fixation count | dNoError | 1 | 2127 | 2315.32 | <.0001 |
| Fixation count | dErrSem | 1 | 2127 | 645.11 | <.0001 |
| Fixation count | dErrSyn | 1 | 2127 | 1415.46 | <.0001 |
| Fixation count | dErrSyn2 | 1 | 688 | 30.39 | <.0001 |
| Fixation count | Answer | 1 | 2127 | 3.2 | 0.0739 |
| Fixation duration (ms) | Sex | 1 | 2128 | 11.9 | 0.0006 |
| Fixation duration (ms) | LogAge | 1 | 2127 | 5.56 | 0.0184 |
| Fixation duration (ms) | Expertise | 1 | 2128 | 3.38 | 0.066 |
| Fixation duration (ms) | dPerformance | 1 | 2128 | 0.55 | 0.4583 |
| Fixation duration (ms) | dGoodExpert | 1 | 1018 | 0.07 | 0.7959 |
| Fixation duration (ms) | dNoError | 1 | 2127 | 801.78 | <.0001 |
| Fixation duration (ms) | dErrSem | 1 | 2127 | 511.65 | <.0001 |
| Fixation duration (ms) | dErrSyn | 1 | 2127 | 679.23 | <.0001 |
| Fixation duration (ms) | dErrSyn2 | 1 | 688 | 24.93 | <.0001 |
| Fixation duration (ms) | Answer | 1 | 2127 | 0.23 | 0.633 |
| Fixation duration (%) | Sex | 1 | 2128 | 0.67 | 0.4127 |
| Fixation duration (%) | LogAge | 1 | 2127 | 1.31 | 0.2519 |
| Fixation duration (%) | Expertise | 1 | 2128 | 0.01 | 0.9231 |
| Fixation duration (%) | dPerformance | 1 | 2128 | 0.32 | 0.5734 |
| Fixation duration (%) | dGoodExpert | 1 | 1018 | 0.03 | 0.8678 |
| Fixation duration (%) | dNoError | 1 | 2127 | 2347.2 | <.0001 |
| Fixation duration (%) | dErrSem | 1 | 2127 | 476.09 | <.0001 |
| Fixation duration (%) | dErrSyn | 1 | 2127 | 418.98 | <.0001 |
| Fixation duration (%) | dErrSyn2 | 1 | 688 | 0 | 0.9796 |
| Fixation duration (%) | Answer | 1 | 2127 | 55.68 | <.0001 |
| Perf_Total | Sex | 1 | 28 | 3.69 | 0.065 |
| Perf_Total | LogAge | 1 | 28 | 2.03 | 0.1658 |
| Perf_Total | Expertise | 1 | 28 | 0 | 0.9642 |
| Perf_Total | dPerformance | 1 | 28 | 13.06 | 0.0012 |
| Perf_Total | dGoodExpert | 1 | 13 | 4.92 | 0.0449 |
| Perf_NoError | Sex | 1 | 28 | 8.48 | 0.007 |
| Perf_NoError | LogAge | 1 | 28 | 4.11 | 0.0523 |
| Perf_NoError | Expertise | 1 | 28 | 4.49 | 0.0432 |
| Perf_NoError | dPerformance | 1 | 28 | 10.89 | 0.0026 |
| Perf_NoError | dGoodExpert | 1 | 13 | 11.32 | 0.0051 |
| Perf_Sem | Sex | 1 | 28 | 0 | 0.9849 |
| Perf_Sem | LogAge | 1 | 28 | 0.26 | 0.613 |
| Perf_Sem | Expertise | 1 | 28 | 0.69 | 0.4122 |
| Perf_Sem | dPerformance | 1 | 28 | 4.11 | 0.0522 |
| Perf_Sem | dGoodExpert | 1 | 13 | 0.06 | 0.8176 |
| Perf_syn | Sex | 1 | 28 | 1.51 | 0.2291 |
| Perf_syn | LogAge | 1 | 28 | 0.87 | 0.3595 |
| Perf_syn | Expertise | 1 | 28 | 1 | 0.3263 |
| Perf_syn | dPerformance | 1 | 28 | 3.35 | 0.0777 |
| Perf_syn | dGoodExpert | 1 | 13 | 1.11 | 0.3103 |
| Perf_syn2 | Sex | 1 | 28 | 0.51 | 0.4819 |
| Perf_syn2 | LogAge | 1 | 28 | 0.11 | 0.7404 |
| Perf_syn2 | Expertise | 1 | 28 | 0.28 | 0.5998 |
| Perf_syn2 | dPerformance | 1 | 28 | 0.3 | 0.5884 |
| Perf_syn2 | dGoodExpert | 1 | 13 | 0 | 0.9465 |
| Perf_syn3 | Sex | 1 | 28 | 6.09 | 0.0199 |
| Perf_syn3 | LogAge | 1 | 28 | 1.28 | 0.2677 |
| Perf_syn3 | Expertise | 1 | 28 | 1.22 | 0.2791 |
| Perf_syn3 | dPerformance | 1 | 28 | 5.61 | 0.025 |
| Perf_syn3 | dGoodExpert | 1 | 13 | 2.6 | 0.1307 |
| Answer | Sex | 1 | 2128 | 3.2 | 0.0736 |
| Answer | LogAge | 1 | 2127 | 1.31 | 0.2521 |
| Answer | Expertise | 1 | 2128 | 0 | 0.9716 |
| Answer | dPerformance | 1 | 2128 | 12.95 | 0.0003 |
| Answer | dGoodExpert | 1 | 1018 | 4.09 | 0.0435 |
| Answer | dNoError | 1 | 2127 | 2.07 | 0.1499 |
| Answer | dErrSem | 1 | 2127 | 10.71 | 0.0011 |
| Answer | dErrSyn | 1 | 2127 | 1.78 | 0.1822 |
| Answer | dErrSyn2 | 1 | 688 | 0.21 | 0.6435 |
| Input | Sex | 1 | 508 | 4.66 | 0.0314 |
| Input | LogAge | 1 | 507 | 1.22 | 0.2698 |
| Input | Expertise | 1 | 508 | 2.9 | 0.0893 |
| Input | dPerformance | 1 | 508 | 2.48 | 0.1157 |
| Input | dGoodExpert | 1 | 239 | 5.75 | 0.0173 |
| Input | dNoError | 1 | 507 | 34.42 | <.0001 |
| Input | dErrSem | 1 | 507 | 49.15 | <.0001 |
| Input | dErrSyn | 1 | 507 | 5.13 | 0.0239 |
| Input | dErrSyn2 | 1 | 130 | 11.63 | 0.0009 |

Note: Repeated measures (except for performance measures) ANOVA based on type 3 sum of squares calculated using Proc Glimmix in SAS

**ANOVA Statistics for Table 2**

| Dependent Variable | Effect | NumDF | DenDF | FValue | ProbF |
| --- | --- | --- | --- | --- | --- |
| Total view Time | dNoError | 1 | 1017 | 72.48 | <.0001 |
| Total view Time | dErrSem | 1 | 1017 | 1.25 | 0.2639 |
| Total view Time | dErrSyn | 1 | 1017 | 43.99 | <.0001 |
| Total view Time | dErrSyn2 | 1 | 328 | 13.26 | 0.0003 |
| Fixation count | dNoError | 1 | 1017 | 1244.76 | <.0001 |
| Fixation count | dErrSem | 1 | 1017 | 265 | <.0001 |
| Fixation count | dErrSyn | 1 | 1017 | 686.77 | <.0001 |
| Fixation count | dErrSyn2 | 1 | 328 | 35.38 | <.0001 |
| Fixation duration (ms) | dNoError | 1 | 1017 | 408.84 | <.0001 |
| Fixation duration (ms) | dErrSem | 1 | 1017 | 237.33 | <.0001 |
| Fixation duration (ms) | dErrSyn | 1 | 1017 | 343.19 | <.0001 |
| Fixation duration (ms) | dErrSyn2 | 1 | 328 | 43.44 | <.0001 |
| Fixation duration (%) | dNoError | 1 | 1017 | 835.84 | <.0001 |
| Fixation duration (%) | dErrSem | 1 | 1017 | 227.96 | <.0001 |
| Fixation duration (%) | dErrSyn | 1 | 1017 | 157.5 | <.0001 |
| Fixation duration (%) | dErrSyn2 | 1 | 328 | 2.95 | 0.0869 |

Note: Repeated measures (except for performance measures) ANOVA based on type 3 sum of squares calculated using Proc Glimmix in SAS

**ANOVA Statistics for Table 3**

| Dependent Variable | Effect | NumDF | DenDF | FValue | ProbF |
| --- | --- | --- | --- | --- | --- |
| Total view Time | dNoError | 1 | 560 | 40.26 | <.0001 |
| Total view Time | dErrSem | 1 | 560 | 4.47 | 0.0349 |
| Total view Time | dErrSyn | 1 | 560 | 29.96 | <.0001 |
| Total view Time | dErrSyn2 | 1 | 180 | 14.74 | 0.0002 |
| Fixation count | dNoError | 1 | 560 | 603.8 | <.0001 |
| Fixation count | dErrSem | 1 | 560 | 102.44 | <.0001 |
| Fixation count | dErrSyn | 1 | 560 | 421.73 | <.0001 |
| Fixation count | dErrSyn2 | 1 | 180 | 29.36 | <.0001 |
| Fixation duration (ms) | dNoError | 1 | 560 | 280.94 | <.0001 |
| Fixation duration (ms) | dErrSem | 1 | 560 | 101.51 | <.0001 |
| Fixation duration (ms) | dErrSyn | 1 | 560 | 320.13 | <.0001 |
| Fixation duration (ms) | dErrSyn2 | 1 | 180 | 27.97 | <.0001 |
| Fixation duration (%) | dNoError | 1 | 560 | 556.49 | <.0001 |
| Fixation duration (%) | dErrSem | 1 | 560 | 119.04 | <.0001 |
| Fixation duration (%) | dErrSyn | 1 | 560 | 108.26 | <.0001 |
| Fixation duration (%) | dErrSyn2 | 1 | 180 | 4.42 | 0.0369 |

Note: Repeated measures (except for performance measures) ANOVA based on type 3 sum of squares calculated using Proc Glimmix in SAS

**ANOVA Statistics for Table 4**

| Dependent Variable | Effect | NumDF | DenDF | FValue | ProbF |
| --- | --- | --- | --- | --- | --- |
| Input | Expertise | 1 | 509 | 2.88 | 0.0903 |
| Input | dGoodExpert | 1 | 240 | 5.4 | 0.021 |
| Input (Error_type=1) | Expertise | 1 | 189 | 0.12 | 0.7346 |
| Input (Error_type=1) | dGoodExpert | 1 | 82 | 5.17 | 0.0256 |
| Input (Error_type=2) | Expertise | 1 | 38 | 8.12 | 0.007 |
| Input (Error_type=2) | dGoodExpert | 1 | 15 | 1.04 | 0.3229 |
| Input (Error_type=3) | Expertise | 1 | 72 | 2.09 | 0.153 |
| Input (Error_type=3) | dGoodExpert | 1 | 27 | 0.79 | 0.3821 |

Note: Repeated measures (except for performance measures) ANOVA based on type 3 sum of squares calculated using Proc Glimmix in SAS

## 
